# Vestibulo-Ocular Reflex Function and Its Impact on Postural Stability and Quality of Life in Cochlear Implant Recipients: A Cross-Sectional Study

**DOI:** 10.3390/life15030499

**Published:** 2025-03-20

**Authors:** Khalid A. Alahmari, Sarah Alshehri

**Affiliations:** 1 Program of Physical Therapy, Department of Medical Rehabilitation Sciences, College of Applied Medical Sciences, King Khalid University, Abha 61421, Saudi Arabia; kahmarie@kku.edu.sa; 2 Otology and Neurotology, Department of Surgery, College of Medicine, King Khalid University, Abha 61421, Saudi Arabia

**Keywords:** vestibulo-ocular reflex, vHIT, postural stability, cochlear implants, quality of life, semicircular canals

## Abstract

Background/Objectives: Vestibulo-ocular reflex (VOR) function, measured by the video head impulse test (vHIT) gains, plays a crucial role in postural stability and quality of life. Cochlear implant recipients often experience vestibular dysfunction, but its relationship with balance and patient-reported outcomes remains underexplored. This study aimed to (1) evaluate the relationship between vHIT gains and postural stability in cochlear implant recipients; (2) assess the impact of vHIT gains on quality-of-life metrics; and (3) identify key predictors of postural stability, including vHIT gains and demographic/clinical characteristics. Methods: This cross-sectional study was conducted between August 2023 and February 2024 and included 46 participants that comprised cochlear implant recipients and age-matched normal hearers who underwent the vHIT for lateral, anterior, and posterior semicircular canal function. Postural stability was assessed using dynamic posturography, and quality of life was measured using the Short Form-36 (SF-36). Multiple linear regression and correlation analyses were performed. Results: The vHIT gains demonstrated significant positive correlations with postural stability, with the lateral canal showing the strongest association (r = 0.742, *p* = 0.001), followed by the posterior (r = 0.701, *p* = 0.003) and anterior canals (r = 0.684, *p* = 0.005). A multiple regression analysis identified the lateral canal as the most significant predictor of postural stability (β = 0.512, *p* = 0.001, adjusted R^2^ = 0.47). Quality-of-life metrics were inversely correlated with the vHIT gains, particularly in the posterior canal (r = −0.712, *p* = 0.002), which explained 43–51% of the variance. Conclusions: This study highlighted the lateral semicircular canal as the primary determinant of postural stability in cochlear implant recipients, underscoring the importance of vestibular assessments in optimizing balance and functional outcomes.

## 1. Introduction

The vestibular system plays a critical role in maintaining postural stability and coordinating eye movements through the vestibulo-ocular reflex (VOR) [1]. This intricate mechanism enables individuals to maintain balance and clear vision during head movements, relying heavily on the semicircular canals and otolith organs [1]. Any disruption in vestibular function can impair balance and stability, significantly impacting daily activities and overall quality of life [2]. In cochlear implant recipients, vestibular dysfunction is particularly relevant, as implantation procedures and underlying auditory pathologies can compromise the vestibular integrity [3]. Quality of life impairment is a well-documented consequence of vestibular dysfunction, with significant effects observed in various vestibular disorders [4]. Beyond impairments in balance and spatial orientation, vestibular dysfunction is often associated with psychiatric symptoms, including anxiety and depression, further exacerbating the overall burden on well-being [5]. Previous research, including a meta-analysis by Ibrahim et al. [6], demonstrated that cochlear implantation can induce vestibular dysfunction in a subset of recipients, with varying degrees of impairment across semicircular canals. This highlights the need for routine vestibular assessments to identify at-risk patients and guide rehabilitation strategies. Given the interplay between vestibular deficits and psychological health, assessing the quality of life in cochlear implant recipients with vestibular dysfunction is essential to fully understand the impact of these impairments [5].

The video head impulse test (vHIT) is a valuable tool for assessing vestibular function, as it employs physiological high-frequency stimuli to evaluate the semicircular canal function [7]. Unlike caloric testing, which is limited to assessing the lateral canal under low-frequency conditions, the vHIT provides a comprehensive assessment of all six semicircular canals, allowing for a more complete evaluation of the vestibular integrity [8]. Cochlear implantation is a widely accepted intervention for individuals with severe-to-profound sensorineural hearing loss who derive limited benefit from hearing aids [9]. By bypassing the damaged cochlear structures and directly stimulating the auditory nerve, cochlear implants restore auditory perception, significantly improving communication and overall quality of life. However, cochlear implantation may also impact vestibular function due to the anatomical proximity of the auditory and vestibular structures, necessitating careful evaluation of vestibular integrity in these patients [10].

Postural stability is a multifactorial process influenced by sensory input, motor control, and central processing [11]. In addition to vestibular function, factors such as age, body mass index (BMI), duration of hearing loss, and implant use may contribute to balance outcomes [12]. Identifying key predictors of postural stability can provide valuable insights into the unique challenges faced by cochlear implant recipients and guide tailored interventions [13]. While some studies examined the relationship between demographic and clinical factors and postural stability, the integration of vHIT metrics into this analysis represents a novel approach to understanding balance control in this population [14,15,16].

Despite advancements in cochlear implant technology and rehabilitation, there remains a limited understanding of the interplay between vestibular function, postural stability, and quality of life in cochlear implant recipients [17,18]. Previous studies primarily focused on vestibular deficits following implantation or general measures of balance, with minimal attention to the predictive value of vHIT metrics and the contribution of demographic and clinical factors. This research aimed to address these gaps by providing a comprehensive analysis of vestibular function and its clinical implications, emphasizing the importance of integrating vestibular assessments into routine care for cochlear implant users. The objectives of this study were threefold: (1) to evaluate the relationship between vHIT gains and postural stability in cochlear implant recipients, (2) to assess the impact of vHIT gains on quality-of-life metrics, and (3) to identify key predictors of postural stability, including vHIT gains and demographic/clinical characteristics.

## 2. Materials and Methods

### 2.1. Settings, Ethics, and Study Design

This prospective cross-sectional study was conducted between August 2023 and February 2024 at the Department of Otolaryngology, KKU Medical City, Abha, a center with specialized facilities for cochlear implantation and vestibular evaluation. Ethical approval for this study was obtained from the institutional review board of DSR, KKU (REC# 239-2023), on 23 July 2023, which ensured compliance with all the applicable guidelines and regulations. Informed written consent was obtained from all the participants before their enrollment in this study, and this research adhered to the ethical principles set forth in the Declaration of Helsinki. Data were collected and stored securely, and strict confidentiality was maintained throughout this study. The control normal hearing group was included solely to establish baseline demographic comparability and ensure that any observed effects on postural stability and VOR function were specific to the CI recipients. Their data were not included in the correlational or regression analyses.

### 2.2. Participants

**Figure 1:**
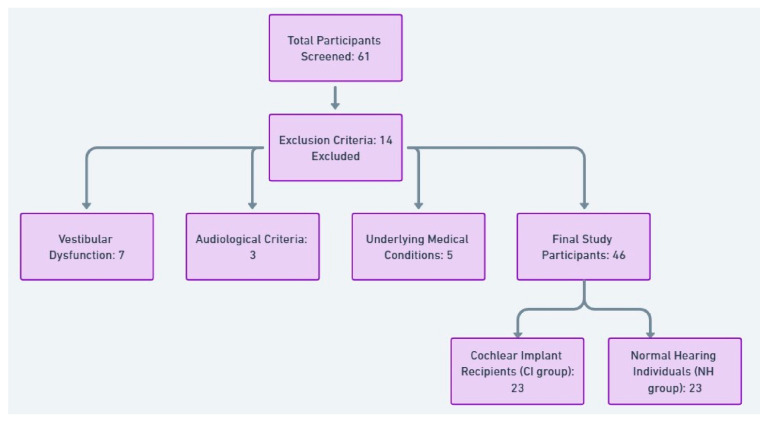
Participant selection flowchart depicting screening, exclusion, and final study groups.

A total of 61 participants were initially screened for eligibility. Fourteen cases were excluded due to vestibular dysfunction (*n* = 7), audiological criteria (*n* = 3), or underlying medical conditions (*n* = 5), which left 46 participants for this study. The remaining individuals were divided into two groups: 23 cochlear implant recipients (CI group) and 23 age-matched normal-hearing individuals (NH group). The NH group was included solely for demographic comparability and was not part of the inferential statistical analyses. Figure 1 provides a visual representation of the participant selection process.

Cochlear implantation was performed based on internationally recognized criteria, including postlingual severe-to-profound sensorineural hearing loss (SNHL) confirmed by pure-tone audiometry (PTA) and limited benefit from hearing aids [19]. Candidates underwent comprehensive audiological and medical evaluations to assess their suitability for implantation. The CI group consisted of adults aged 18–70 years with a documented history of postlingual SNHL and at least one year of stable cochlear implant use. Stability was defined by the absence of significant post-implantation complications and consistent audiological benefit, which ensured reliable long-term outcome assessment. Both unilateral and bilateral CI recipients were included in this study. The participants were required to have measurable vestibular function in at least one semicircular canal, as assessed by the vHIT (EyeSeeCam vHIT, Interacoustics, Denmark). Additionally, all participants completed dynamic posturography and the Short Form-36 (SF-36) quality-of-life questionnaire. The NH group comprised adults aged 18–70 years with bilateral normal hearing thresholds (≤25 dB HL at 0.25–8 kHz in both ears, confirmed via PTA); no history of auditory or vestibular disorders, middle ear infections, or balance-related issues; and normal vestibular function confirmed through the vHIT and dynamic posturography. The participants from both groups were excluded if they had neurological, musculoskeletal, or systemic conditions that affected their balance (e.g., stroke, Parkinson’s disease, advanced arthritis); a history of middle ear surgery (other than cochlear implantation for the CI group) or recent vestibular disorders (Ménière’s disease, vestibular neuritis) within the last six months; uncontrolled diabetes, cardiovascular disease, or autoimmune inner ear disorders known to affect vestibular function; use of vestibular-suppressant medications within 72 h of testing; or cognitive impairments or psychiatric conditions that could interfere with test participation or questionnaire completion. Additional exclusion criteria specific to the CI group included recent cochlear implant failure or complications affecting device functionality, while for the NH group, participants with undetected subclinical vestibular dysfunction (determined by abnormal vHIT results) were excluded.

The NH group was included solely to establish baseline demographic comparability. Their data were not used in the correlation, regression, or predictive analyses that assessed vestibular function, postural stability, or quality of life. All inferential statistical models, including the correlation and regression analyses, were performed exclusively within the CI group. The NH group data were excluded from inferential analyses because this study aimed to assess how the vHIT gains influenced postural stability and quality of life, specifically in CI recipients, where vestibular function is more clinically relevant.

### 2.3. Variables

The primary variables of interest in this study included VOR function, postural stability, and quality of life.

#### 2.3.1. Audiological Testing and Vestibular Assessment

Audiological testing included pure-tone audiometry (PTA) (GSI Audiostar Pro, Grason-Stadler Inc., Eden Prairie, MN, USA), which assessed the air and bone conduction thresholds at 0.25, 0.5, 1, 2, 4, and 8 kHz, along with speech audiometry to measure word recognition scores in quiet conditions. Only postlingual cochlear implant recipients were included, which ensured the applicability of PTA for all participants. The test evaluated all six semicircular canals bilaterally and calculated the gain values and asymmetry between sides to provide a comprehensive measure of vestibular function [20]. Both unilateral and bilateral cochlear implant recipients were included in this study, with implant configuration recorded for each participant to assess its potential influence on vestibular function. The vHIT was used to assess the VOR function by applying high-frequency, small-amplitude head impulses to evaluate the semicircular canal responses [21]. Unlike caloric testing, which only evaluates the lateral canal at low frequencies, the vHIT provides a comprehensive, physiological assessment of all six semicircular canals bilaterally [8]. The test was conducted using a high-speed infrared camera system to track the eye velocity in response to passive, unpredictable head movements in the horizontal and vertical planes [22]. Mean VOR gains for the lateral, anterior, and posterior canals on both sides were automatically calculated, with a minimum of ten valid head impulses recorded per canal to ensure reliability. Normal gain values were considered ≥0.8 for the lateral canals and ≥0.7 for the anterior and posterior canals, with lower values indicating potential vestibular dysfunction. The vHIT testing was performed by an experienced neurotologist to ensure consistency and minimize interobserver variability.

The participants were instructed to maintain steady fixation on a small, stationary target placed directly in front of them at eye level throughout the test. Rapid, passive head impulses were delivered manually by the examiner, with angular displacements of approximately 10–20° in each direction. These impulses were performed unpredictably in terms of timing and direction to prevent anticipatory eye movements. Testing was conducted for each of the three semicircular canals. For the lateral canal, the head was rotated quickly to the left and right in the horizontal plane. For the anterior canal, the head was tilted downward and rotated upward diagonally in the direction of the canal being tested. For the posterior canal, the head was tilted upward and rotated downward diagonally toward the corresponding canal.

The eye and head movements were recorded simultaneously using high-speed infrared cameras with a frame rate of at least 250 Hz to ensure the precise tracking of rapid movements. VOR gains were calculated automatically by the system as the ratio of the velocity of the participant’s eye movement to the velocity of their head movement during each impulse. A minimum of 10 valid head impulses were performed for each canal in each direction to ensure reliability and reduce variability. Outlier impulses, such as those with excessive head movement artifacts, were excluded automatically by the system’s software, and the mean VOR gain for each canal was calculated based on the remaining valid impulses. Care was taken to deliver head impulses within a safe range of motion to avoid discomfort or injury, and participants were provided with breaks as needed to prevent fatigue that could affect the accuracy of the test.

The primary output from the vHIT system consisted of the mean VOR gains for the lateral, anterior, and posterior canals, which served as the independent variables in the analysis. This standardized and replicable vHIT protocol ensured high-quality data collection that provided a comprehensive evaluation of semicircular canal function in all participants.

#### 2.3.2. Assessment of Postural Stability

Postural stability, the primary outcome of this study, was assessed using standardized dynamic posturography tests (Iso-free, Techno-Body, Dalmine, Bergamo, Italy), which provide a reliable and objective measure of an individual’s ability to maintain balance under varying conditions. The participants were tested in a controlled laboratory environment to ensure consistent procedures and minimize external influences. The assessment involved a series of balance tasks performed under different surface and sensory conditions to evaluate the participants’ postural control mechanisms comprehensively. These conditions included standing on stable and unstable surfaces, such as a firm platform and a foam surface, and tasks performed with eyes open and closed to challenge sensory integration processes that involved visual, vestibular, and somatosensory systems. Each participant was instructed to stand upright on the designated surface with their feet shoulder-width apart and maintain their balance for a predetermined duration during each task. The test administrator provided clear instructions before each task and observed the participants to ensure adherence to the protocol. Movements of the center of pressure and sway patterns were recorded using a force plate embedded in the testing platform to capture precise data on the participant’s ability to maintain balance. The tasks were progressively challenging and designed to assess the participants’ adaptive responses to varying degrees of difficulty and sensory input reliance. The data from all tasks were processed and analyzed to compute a composite postural stability score for each participant. This score reflected the overall balance performance and was derived from quantitative metrics, such as the sway velocity, sway area, and the ability to maintain equilibrium under different testing conditions. These composite scores served as the primary dependent variable in this study and were used to evaluate the relationship between the vestibular function, as measured by the vHIT gains, and the balance control.

#### 2.3.3. Assessment of Quality of Life

Quality of life, a key outcome of this study, was evaluated using the Short Form-36 (SF-36) questionnaire [23], a widely validated and reliable patient-reported outcome measure. The SF-36 was chosen due to its broad applicability in assessing general health-related quality of life across physical, mental, and functional domains [23]. While CI-specific QoL tools, such as the Nijmegen Cochlear Implant Questionnaire (NCIQ), focus primarily on auditory-related outcomes, SF-36 enables a comprehensive evaluation of postural stability and vestibular dysfunction impacts, which were key aspects of this study [24]. Additionally, SF-36 allows for comparisons with general population norms and existing CI research, ensuring consistency with prior literature. To ensure cultural and linguistic appropriateness, the validated version of the SF-36 in Arabic, the primary language of this study population, was used to maintain its reliability for assessing patient-reported outcomes in this context. To facilitate the analysis, composite scores were calculated for physical health domains and mental health domains, which provided an overall representation of the participant’s quality of life. These composite scores served as dependent variables and were analyzed in relation to the VOR function, as measured by the vHIT gains.

#### 2.3.4. Demographic and Clinical Variables

The demographic and clinical variables included age, body mass index (BMI), the duration of hearing loss, and the duration of cochlear implant use. These data were obtained from patient medical records and standardized questionnaires completed during clinical visits. Age and BMI were recorded as continuous variables, while the durations of hearing loss and implant use were reported in years. These variables were included as potential confounders in the analysis to account for their influence on the postural stability and quality of life outcomes.

### 2.4. Sample Size Estimation

The sample size for this study was determined using G*Power statistical software (version 3.1.9.7, Heinrich Heine University, Düsseldorf, Germany) to ensure sufficient power to detect significant relationships between the VOR function, postural stability, and quality of life. An a priori power analysis was conducted for a multiple linear regression model with three predictors (vHIT gains for the lateral, anterior, and posterior semicircular canals). Based on a medium effect size (Cohen’s f^2^ = 0.15), a significance level (α) of 0.05, and a power (1-β) of 0.80, the minimum required sample size was calculated to be 43 participants. To account for potential dropouts or incomplete data, an additional 7% was added, bringing the final target sample size to 46 participants.

### 2.5. Data Analysis

Data were analyzed using SPSS software (version 24.0, IBM Corp., Armonk, NY, USA). Descriptive statistics were used to summarize the demographic and clinical characteristics of the participants, with continuous variables presented as the mean ± standard deviation (SD) and categorical variables as frequencies and percentages. The normality of the data was confirmed using the Shapiro–Wilk test. Comparative analyses between the CI and NH groups were limited to the baseline demographic characteristics and assessed using independent samples t-tests and chi-square tests. All correlation and regression analyses were conducted exclusively within the CI group. Parametric statistical tests were applied to evaluate the relationships between the variables. Independent samples t-tests were used to compare the vHIT gains, postural stability scores, and quality-of-life metrics between the cochlear implant recipients and normal-hearing controls. Pearson’s correlation coefficient was employed to assess the strength and direction of the relationships between the vHIT gains, postural stability, and quality-of-life scores. Multiple linear regression analyses were conducted to identify predictors of postural stability and quality of life by incorporating vHIT gains and relevant demographic and clinical variables as independent factors. A *p*-value of <0.05 was considered statistically significant for all the analyses.

## 3. Results

Table 1 presents the demographic and clinical characteristics of the cochlear implant recipients (CI group) and normal-hearing controls (NH group). The NH group was included solely for baseline demographic comparability to ensure that the observed differences in postural stability and VOR function were specific to the CI group. No comparative statistical analyses were conducted beyond the baseline verification. Independent samples *t*-tests were used for continuous variables (age, BMI, duration of hearing loss, hearing thresholds, postural stability scores, and quality-of-life metrics), while chi-square tests were applied to categorical variables (sex distribution and comorbidities). No significant differences were found between the groups. The duration of hearing loss referred exclusively to postlingual cases to ensure comparability. Hearing thresholds were assessed via pure-tone audiometry (PTA) by measuring the air and bone conduction across standard frequencies (0.25–8 kHz). The vHIT values represent post-implantation assessments and are reported as the mean ± standard deviation (SD). The comorbidities included hypertension, diabetes, cardiovascular diseases, and musculoskeletal disorders.

Parameters such as age, BMI, and hearing thresholds, both pre- and post-implant, exhibited closely aligned mean values with small effect sizes (Cohen’s d ranged from −0.132 to 0.125), indicating minimal practical differences. Similarly, the vHIT gains, postural stability scores, and quality-of-life metrics showed negligible differences between the groups, as reflected by *p*-values > 0.05 and small effect sizes.

**Figure 2:**
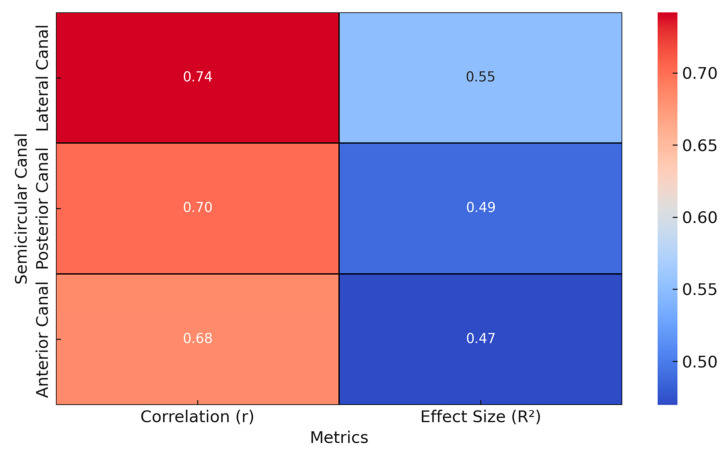
Heatmap illustrating the correlation coefficients (r values) and effect sizes (R^2^) between the video head impulse test (vHIT) gains for the lateral, anterior, and posterior semicircular canals and the postural stability in the cochlear implant recipients. All correlation analyses were performed exclusively within the CI group.

The correlation analysis between the vHIT gains and postural stability in the cochlear implant recipients revealed significant positive associations across all semicircular canals, with the lateral canal showing the strongest correlation (r = 0.742, *p* = 0.001), followed by the posterior (r = 0.701, *p* = 0.003) and anterior canals (r = 0.684, *p* = 0.005) (Table 2 and Figure 2). The coefficients of determination (R^2^) indicate that the vHIT gains explained a substantial proportion of the variance in postural stability, which ranged from 47% to 55%.

Lower vHIT gains, indicating reduced vestibular function, were associated with a diminished quality of life due to increased balance difficulties, higher fall risk, and reduced independence in daily activities.

The multivariate regression analysis (Table 3) demonstrated that the vHIT gains in all semicircular canals significantly predicted the postural stability, where the lateral canal exhibited the strongest effect (β = 0.48, *p* = 0.002).

The anterior and posterior canals also contributed significantly (β = 0.39, *p* = 0.005; β = 0.42, *p* = 0.004, respectively). Age and the duration of hearing loss negatively influenced the postural stability (β = −0.27, *p* = 0.018; β = −0.21, *p* = 0.034), whereas longer implant use was positively associated with better stability (β = 0.31, *p* = 0.008). A group comparison indicated that the CI recipients had significantly lower postural stability than the NH controls (β = −0.62, *p* = 0.001). The BMI did not significantly impact the postural stability (*p* = 0.127). The model explained 51% of the variance in the postural stability, emphasizing the critical role of vestibular function in balance among the CI recipients. Notably, the strong negative β coefficient for the CI group (−0.62, *p* = 0.001) confirmed that the CI recipients exhibited distinct balance challenges compared with the NH individuals, further justifying the need for targeted vestibular assessments.

**Figure 3:**
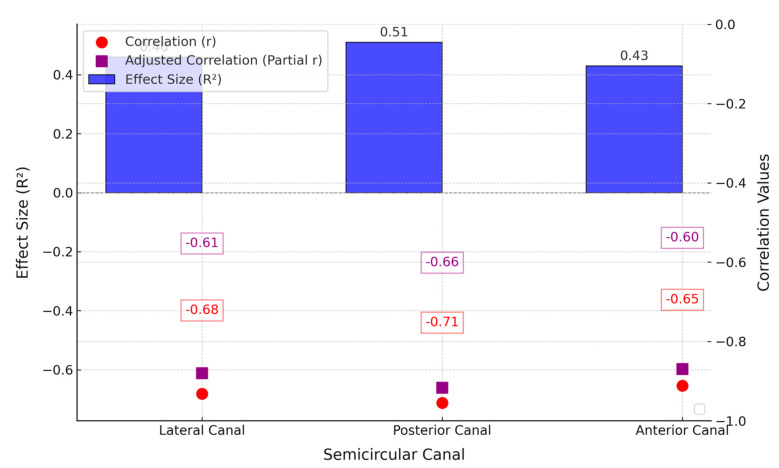
Scatterplot and effect size analysis illustrating the relationship between the semicircular canal function (vHIT gains) and quality of life (SF-36 scores) in the cochlear implant recipients. All correlations were performed exclusively within the CI group.

The analysis of correlations between the vHIT gains and quality-of-life metrics in the cochlear implant recipients revealed significant negative associations for all the semicircular canals, with the posterior canal showing the strongest correlation (r = −0.712, *p* = 0.002), followed by the lateral (r = −0.681, *p* = 0.004) and anterior canals (r = −0.654, *p* = 0.007) (Table 4 and Figure 3).

The adjusted correlations (partial r) remained statistically significant after controlling for the potential confounders, further supporting the inverse relationship between the vestibular function and quality of life. The effect sizes (R^2^) indicate that the vHIT gains accounted for 43% to 51% of the variance in the quality of life, suggesting a meaningful impact of vestibular dysfunction on the patient-reported outcomes.

**Figure 4:**
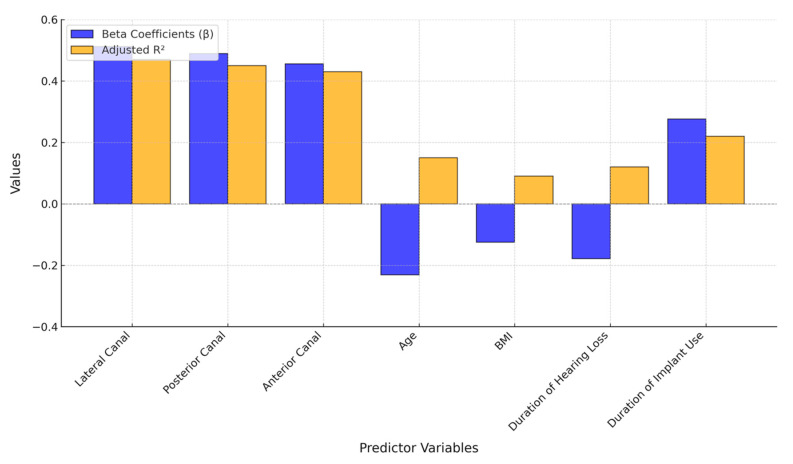
Regression analysis of predictors of postural stability in cochlear implant recipients: beta coefficients and adjusted R^2^ values.

The regression analysis identified the vHIT gains as significant predictors of postural stability, with the lateral canal demonstrating the strongest association (β = 0.512, *p* = 0.001), followed by the posterior (β = 0.489, *p* = 0.003) and anterior canals (β = 0.456, *p* = 0.005) (Table 5 and Figure 4).

These findings suggest that the semicircular canal function plays a crucial role in maintaining balance, particularly in cochlear implant recipients who may rely more on vestibular input for postural control. Among the demographic factors, age (β = −0.231, *p* = 0.031) and duration of hearing loss (β = −0.178, *p* = 0.043) were negatively associated with postural stability, while longer implant use (β = 0.276, *p* = 0.007) was positively correlated, suggesting that adaptive mechanisms may develop over time.

## 4. Discussion

This study examined the relationship between vestibulo-ocular reflex (VOR) function, postural stability, and quality of life in cochlear implant (CI) recipients. Statistical analyses were conducted exclusively within the CI group, while the normal-hearing (NH) group served as a demographic baseline reference. The findings reveal that VOR function, as measured by the vHIT gains, was a key determinant of postural stability and significantly influenced the quality of life. Regression analyses identified additional contributing factors, including age and implant duration. An inverse relationship between the VOR function and quality of life suggests that vestibular dysfunction negatively impacted the patient-reported outcomes, emphasizing the need for routine vestibular assessments in CI management.

The results of this study indicate that the cochlear implant recipients and control participants exhibited no significant differences across demographic and clinical characteristics, which ensured robust baseline comparability. The lack of differences in parameters such as age, BMI, hearing thresholds, and vestibulo-ocular reflex (vHIT) gains highlights the well-matched control group, which minimized confounding influences. The observed positive relationship between the vHIT gains and postural stability emphasized the integral role of semicircular canal function in maintaining balance, particularly in populations reliant on precise vestibular input due to auditory interventions [25]. The inverse relationship between the vHIT gains and quality-of-life metrics further underscored the complex interplay between vestibular function and the patient-reported outcomes [26]. Higher vHIT gains indicate better vestibular integrity, which enhances postural stability and reduces fall risk, allowing for greater mobility and independence [25]. Conversely, lower vHIT gains suggest vestibular dysfunction, often leading to dizziness, balance impairments, and functional limitations that restrict daily activities and negatively impact quality of life [27]. While higher vHIT gains suggest preserved vestibular integrity, the associated quality-of-life implications may reflect broader impacts of vestibular dysfunction on daily activities and well-being, a finding that warrants further investigation [28]. These findings align with existing literature emphasizing the significance of vestibular function in postural control and quality of life. Vaz et al. [28] and Sosna-Duranowska et al. [28] reported that cochlear implant recipients often demonstrate varying degrees of vestibular function preservation, which directly correlates with their ability to maintain balance and stability. Additionally, previous studies by Yang [29] and Castellucci et al. [30] identified semicircular canal deficits as critical determinants of functional outcomes in individuals with vestibular impairment, with significant associations observed across all canals. The horizontal canal in particular was highlighted due to its primary role in stabilizing gaze and maintaining balance, which aligns with our findings, which demonstrate its strongest correlation with postural stability [30]. The lack of significant differences in the quality-of-life metrics between the groups is consistent with findings by Andries et al. [31], who suggested that cochlear implantation alone does not adversely affect the overall life satisfaction in recipients with preserved vestibular function. Collectively, the current study corroborates these findings, emphasizing the pivotal role of vestibular evaluation in optimizing clinical outcomes for cochlear implant users.

The significant positive correlations between the vHIT gains and postural stability observed in this study underscore the fundamental role of vestibular function in maintaining balance in cochlear implant recipients. The lateral canal demonstrated the strongest association with postural stability, followed by the posterior and anterior canals, which is consistent with the physiological role of the semicircular canals in detecting angular head motion and providing critical input for balance and gaze stabilization [32]. The substantial proportion of variance in postural stability explained by the vHIT gains (47% to 55%) highlights the direct impact of semicircular canal functionality on postural control [33]. These findings can be attributed to the reliance of cochlear implant recipients on preserved vestibular function to compensate for sensory changes associated with implantation and auditory processing adjustments. Furthermore, the inverse relationship between the vHIT gains and quality-of-life metrics suggests that while preserved vestibular function supports postural control, residual vestibular dysfunction may still impose limitations on daily activities, contributing to a reduced perception of quality of life [25]. These results align with findings from previous studies that emphasize the critical role of vestibular function in functional and patient-reported outcomes. For instance, Lacour et al. [34] reported that semicircular canal function strongly correlates with balance performance in patients with vestibular impairment, with the lateral canal having a predominant role [34]. Similarly, the work of Lacour et al. [34] identified the vestibular system as a key determinant of postural stability and functional mobility in cochlear implant recipients. The inverse correlation between the vHIT gains and quality of life is supported by studies such as those by Wagner et al. [25] and Hall et al. [35], which highlighted the substantial impact of vestibular dysfunction on quality of life, particularly in terms of mobility and independence. Choi et al. [36] further demonstrated that vestibular rehabilitation strategies could improve postural stability and quality of life in this population. Together, these findings reinforce the need for integrated vestibular assessment and management to optimize outcomes for cochlear implant recipients [37].

The regression analysis highlighted the pivotal role of semicircular canal function, as measured by the vHIT gains, in predicting the postural stability among the cochlear implant recipients. The lateral canal emerged as the strongest predictor, followed by the posterior and anterior canals, reflecting the critical contribution of these structures to the integration of vestibular input for balance and spatial orientation [38]. The higher beta coefficients for the semicircular canals suggest that vestibular function is a primary determinant of postural control in this population, likely due to its role in detecting angular head motion and coordinating postural adjustments [32]. Additional predictors, such as age, the duration of hearing loss, and implant use, also demonstrated significant but smaller effects, underscoring the multifactorial nature of postural stability [39]. In contrast, BMI did not significantly impact postural stability, suggesting that vestibular and age-related factors play a more direct role in influencing balance in this cohort [40]. These findings are consistent with prior studies that emphasized the role of vestibular function in maintaining balance and postural control [41]. Allum et al. [41] identified the lateral semicircular canal as a critical contributor to postural stability in patients with vestibular deficits, aligning with the current study’s findings of its strongest predictive value. Similarly, Soto et al. [42] demonstrated that semicircular canal integrity significantly influences the ability to perform balance-related tasks, particularly in populations with auditory prostheses. The impact of age and duration of hearing loss on postural stability has also been reported in studies such as those by Thompson-Harvey et al. [43], who highlighted the cumulative effects of aging and sensory impairment on vestibular function [43]. Furthermore, Ovari et al. [44] observed that the duration of cochlear implant use positively correlates with adaptive improvements in postural stability, consistent with the current findings [44]. These results collectively underscore the importance of assessing and addressing vestibular function to optimize clinical outcomes in cochlear implant recipients.

Our study controlled for major systemic conditions affecting vestibular function, medication use, and other comorbidities that may have influenced postural stability and quality-of-life outcomes [6]. Certain medications, such as vestibular suppressants, antihypertensives, or psychotropic drugs, can alter balance control, while comorbid conditions, like anxiety, depression, and musculoskeletal disorders, may contribute to postural instability. Future research should incorporate a more comprehensive evaluation of these factors to further refine our understanding of their role in vestibular function among cochlear implant recipients. While this study primarily utilized the vHIT to assess semicircular canal function, we acknowledge that incorporating caloric testing and VEMPs could enhance the evaluation of vestibular dysfunction. Caloric testing provides insight into low-frequency responses of the horizontal canal, complementing the vHIT’s high-frequency assessment. Similarly, VEMPs assess otolith organ function, offering additional information on vestibular integrity beyond semicircular canal function. Future studies should integrate these modalities for a more comprehensive understanding of vestibular dysfunction in cochlear implant recipients.

Although the SF-36 provided valuable insights into the overall quality of life of CI recipients, future studies may benefit from incorporating CI-specific QoL tools, such as the NCIQ. While SF-36 evaluates broad functional and health domains, the NCIQ focuses specifically on auditory performance and communication-related challenges. A combined approach utilizing both generic and CI-specific QoL measures could enhance the understanding of cochlear implantation outcomes, particularly in relation to vestibular and balance-related impairments.

### 4.1. Clinical Recommendations

Vestibular assessments should be integrated into routine clinical care to optimize postural stability and quality of life in cochlear implant recipients. Pre-implantation screening with the vHIT, caloric testing, and VEMPs can identify patients at risk of vestibular dysfunction, allowing for tailored counseling and rehabilitation strategies. Post-implantation vestibular evaluations, particularly in cases of dizziness or balance impairments, can help detect functional changes and guide vestibular rehabilitation if needed. Dynamic posturography or balance assessments should be incorporated into follow-up visits to monitor postural stability. Multidisciplinary collaboration between otologists, audiologists, and physical therapists can facilitate early intervention, ensuring that vestibular dysfunction is managed alongside auditory rehabilitation. Future research should establish standardized vestibular screening protocols for CI candidates and recipients to improve functional outcomes.

### 4.2. Limitations and Future Directions of This Study

Despite its significant findings, this study has limitations. The cross-sectional design prevents causal inferences, emphasizing the need for longitudinal research to assess the temporal relationship between vHIT gains, postural stability, and quality of life. While key variables were controlled, unmeasured factors, such as comorbidities, medication use, and psychological influences, may have impacted the results. The sample size, though sufficient for correlation analysis, may limit generalizability, particularly for populations with severe vestibular dysfunction. Future research should incorporate structured screening for medications and comorbidities, explore targeted vestibular rehabilitation, and utilize advanced imaging techniques for a more comprehensive assessment of vestibular function. Given the strong correlation between horizontal semicircular canal function and postural stability, adding caloric testing could enhance the evaluation of vestibular asymmetries and hyperfunction. Expanding this study to diverse populations and longitudinal designs will further refine the understanding of vestibular dysfunction in cochlear implant recipients. Although no significant differences in vestibular impairment were observed between the groups at the baseline, the multivariate regression results demonstrate that the vHIT gains strongly predicted postural stability in the CI recipients, supporting the need for routine vestibular assessments in this population.

## 5. Conclusions

This study demonstrated that VOR function, as measured by the vHIT gains, was a significant predictor of postural stability in the cochlear implant recipients, with the lateral semicircular canal showing the strongest association. Additionally, an inverse relationship was observed between the VOR function and quality-of-life metrics, underscoring the impact of vestibular dysfunction on patient-reported outcomes. Age, the duration of hearing loss, and implant use were identified as secondary predictors of postural stability, highlighting the multifactorial nature of balance control in this population. The findings emphasize the importance of integrating vestibular assessments into the routine management of cochlear implant recipients to address postural and functional challenges and improve overall patient care.

## Figures and Tables

**Table 1:** Demographic and clinical characteristics of cochlear implant recipients and normal-hearing group.

Variable	CI Group Mean ± SD (*n* = 23)	NH Group Mean ± SD (*n* = 23)	*p*-Value	Effect Size (Cohen’s d)
Age (years)	49.75 ± 12.35	48.25 ± 11.75	0.634	0.125
Sex (M/F)	12:11	13:10	0.785	0.094
BMI (kg/m^2^)	24.85 ± 2.95	24.55 ± 3.25	0.523	0.105
Duration of hearing loss (years)	14.95 ± 5.55	16.25 ± 6.10	0.687	−0.132
Pre-implant hearing threshold (dB)	85.50 ± 9.98	84.35 ± 9.75	0.592	0.119
Post-implant hearing threshold (dB)	30.65 ± 6.15	-	-	-
vHIT gain (lateral canal)	0.88 ± 0.11	0.89 ± 0.10	0.512	−0.09
vHIT gain (anterior canal)	0.83 ± 0.12	0.84 ± 0.11	0.601	−0.083
vHIT gain (posterior canal)	0.82 ± 0.13	0.83 ± 0.12	0.745	−0.078
Postural stability score	72.85 ± 8.05	71.95 ± 8.25	0.631	0.11
Quality-of-life score	78.95 ± 6.35	78.35 ± 6.50	0.762	0.09
Duration of implant use (years)	4.20 ± 1.25	4.15 ± 1.30	0.841	0.045
Comorbidities (yes/no)	8/10	7/11	0.764	−0.043

CI group, cochlear implant group; NH group, normal-hearing group; BMI, body mass index; dB, decibel; vHIT, video head impulse test; SD, standard deviation; QoL, quality of life. Values are presented as mean ± SD for continuous variables and frequency (percentage) for categorical variables. Independent samples *t*-tests were used for continuous data, and chi-square tests were applied for categorical data (sex distribution and comorbidities). Statistical significance threshold *p* < 0.05. The NH group was included solely for demographic comparison and not used in inferential analyses that evaluated vestibular function, postural stability, and quality-of-life relationships.

**Table 2:** Correlation between vHIT results and postural stability in cochlear implant recipients.

Variable	Mean ± SD	Correlation with Postural Stability (r)	*p*-Value	95% CI for Correlation (r)	Effect Size (R^2^)
vHIT gain (lateral canal)	0.88 ± 0.11	0.742	0.001	(0.582, 0.856)	0.55
vHIT gain (anterior canal)	0.83 ± 0.13	0.684	0.005	(0.489, 0.817)	0.47
vHIT gain (posterior canal)	0.82 ± 0.12	0.701	0.003	(0.526, 0.835)	0.49

vHIT, video head impulse test; SD, standard deviation; r, Pearson correlation coefficient; CI, confidence interval; R^2^, coefficient of determination. The 95% CIs indicate the upper and lower bounds. The significance level for all correlations was *p* < 0.05.

**Table 3:** Multivariate regression analysis including NH and CI groups.

Predictor Variable	Dependent Variable	Beta Coefficient (β)	95% CI for β (Lower, Upper)	*p*-Value	Adjusted R^2^
vHIT gain (lateral canal)	Postural stability	0.48	(0.32, 0.64)	0.002	0.51
vHIT gain (anterior canal)	Postural stability	0.39	(0.24, 0.54)	0.005	0.51
vHIT gain (posterior canal)	Postural stability	0.42	(0.27, 0.57)	0.004	0.51
Age (years)	Postural stability	−0.27	(−0.42, −0.12)	0.018	0.51
BMI (kg/m^2^)	Postural stability	−0.13	(−0.29, 0.03)	0.127	0.51
Duration of hearing loss (years)	Postural stability	−0.21	(−0.35, −0.07)	0.034	0.51
Duration of implant use (years)	Postural stability	0.31	(0.15, 0.47)	0.008	0.51
Group (NH vs. CI)	Postural stability	−0.62	(−0.81, −0.43)	0.001	0.51

vHIT: video head impulse test; BMI: body mass index; NH: normal-hearing group; CI: cochlear implant group; β: beta coefficient; CI: confidence interval; *p*-value: probability value; adjusted R^2^: adjusted coefficient of determination.

**Table 4:** Correlation between vHIT results and their impact on quality-of-life metrics in cochlear implant recipients.

Variable	Mean ± SD	Correlation with Quality of Life (r)	*p*-Value	95% CI for Correlation (r)	Effect Size (R^2^)	Adjusted Correlation (Partial r)	*p*-Value (Adjusted Correlation)
vHIT gain (lateral canal)	0.88 ± 0.11	−0.681	0.004	(−0.812, −0.482)	0.46	−0.612	0.006
vHIT gain (anterior canal)	0.83 ± 0.13	−0.654	0.007	(−0.784, −0.454)	0.43	−0.598	0.008
vHIT gain (posterior canal)	0.82 ± 0.12	−0.712	0.002	(−0.846, −0.541)	0.51	−0.661	0.003

vHIT, video head impulse test; SD, standard deviation; QoL, quality of life; r, Pearson correlation coefficient; CI, confidence interval; R^2^, coefficient of determination; partial r, adjusted Pearson correlation. The 95% CIs indicate the upper and lower bounds. The significance level for all correlations was *p* < 0.05.

**Table 5:** Regression models that predicted postural stability based on vHIT results and potential confounders.

Predictor Variable	Dependent Variable	Beta Coefficient (β)	95% CI for β	*p*-Value	Adjusted R^2^
vHIT gain (lateral canal)	Postural stability	0.512	(0.341, 0.683)	0.001	0.47
vHIT gain (anterior canal)	Postural stability	0.456	(0.278, 0.634)	0.005	0.43
vHIT gain (posterior canal)	Postural stability	0.489	(0.312, 0.666)	0.003	0.45
Age (years)	Postural stability	−0.231	(−0.421, −0.041)	0.031	0.15
BMI (kg/m^2^)	Postural stability	−0.125	(−0.278, 0.028)	0.123	0.09
Duration of hearing loss (years)	Postural stability	−0.178	(−0.345, −0.011)	0.043	0.12
Duration of implant use (years)	Postural stability	0.276	(0.101, 0.451)	0.007	0.22

vHIT, video head impulse test; BMI, body mass index; CI, confidence interval; β, beta coefficient; R^2^, adjusted coefficient of determination. The 95% CIs indicate the upper and lower bounds. The significance level for all predictors was *p* < 0.05.

## Data Availability

The raw data supporting the findings of this study are available at the Zenodo public repository and can be accessed using the following DOI: 10.5281/zenodo.14737981.

## Abbreviations

The following abbreviations are used in this manuscript:

VOR	Vestibulo-ocular reflex
vHIT	Video head impulse test
SF-36	Short Form-36
BMI	Body mass index
QoL	Quality of life
